# Genome wide structural prediction of ABC transporter systems in *Bacillus subtilis*

**DOI:** 10.3389/fmicb.2024.1469915

**Published:** 2024-09-27

**Authors:** Ashwin Mahendran, Benjamin J. Orlando

**Affiliations:** Department of Biochemistry and Molecular Biology, Michigan State University, East Lansing, MI, United States

**Keywords:** ABC transporter, AlphaFold, *Bacillus subtilis*, membrane protein, protein complex

## Abstract

ABC transporters are a diverse superfamily of membrane protein complexes that utilize the binding/hydrolysis of ATP to power substrate movement across biological membranes or perform mechanical work. In bacteria, these transporters play essential roles in biochemical processes ranging from nutrient uptake and protein secretion to antibiotic resistance and cell-wall remodeling. Analysis of the complete genome sequence of the Gram-positive organism *Bacillus subtilis* has previously revealed that ABC transporters comprise the largest family of proteins across the entire genome. Despite the widespread presence of these transporters in *B. subtilis*, relatively few experimental structures of ABC transporters from this organism have been determined. Here we leverage the power of AlphaFold-Multimer to predict the 3-dimensional structure of all potential ABC transporter complexes that have been identified from bioinformatic analysis of the *B. subtilis* genome. We further classify the ABC transporters into discrete classes based on their predicted architecture and the presence or absence of distinct protein domains. The 3-dimensional structure predictions presented here serve as a template to understand the structural and functional diversity of ABC transporter systems in *B. subtilis* and illuminate areas in which further experimental structural validation is warranted.

## Introduction

ATP Binding Cassette (ABC) transporters are a large superfamily of membrane proteins found across all living organisms (Thomas and Tampé, 2020). In bacteria, ABC transporters have long been recognized as critical systems for diverse cellular functions including nutrient uptake (Davidson and Chen, 2004), membrane and cell-wall formation (Bilsing et al., 2023; Rismondo and Schulz, 2021), and virulence or antibiotic resistance (Orelle et al., 2019; Garmory and Titball, 2004; Robertson et al., 2005; Lubelski et al., 2007). As their name suggests, ABC transporters harness the energy of ATP binding/hydrolysis to power transport of molecules across biological membranes. These transport systems have classically been categorized as importers or exporters depending on the direction in which they facilitate substrate transport across lipid membranes (Thomas and Tampé, 2020). However, recent structural and functional studies along with reclassification efforts have revealed alternative unique roles of ABC transporter systems that do not involve movement of molecules across membranes (Thomas et al., 2020). Indeed, some ABC transporter complexes have been shown to perform mechanical work or enzyme activation (Yang et al., 2011), initiate signaling through larger protein complexes (Li et al., 2017; George and Orlando, 2023), or simply extract lipids/proteins from lipid membranes (Sharma et al., 2021; Li et al., 2019).

In 1997, a fundamental breakthrough in microbial genomics was achieved with unveiling of the first complete genome sequence of a Gram-positive organism— the soil bacterium and model organism *Bacillus subtilis* (Kunst et al., 1997). Surprisingly, among the ∼4,100 different protein encoding genes identified in *B. subtilis*, ABC transporters were identified as the largest family of proteins throughout the entire genome. Subsequent bioinformatic work by Quentin, Y. et al. resulted in a comprehensive analysis of the inventory and assembly of ABC transporter systems in *B. subtilis* (Quentin et al., 1999). This analysis revealed that *B. subtilis* contains 78 ABC transporters, including 38 importers and 40 exporters. Since this initial analysis more elaborate classification schemes for ABC transporter systems have been proposed based on newly revealed 3-dimensional structures, topologies, and biological functions (Thomas et al., 2020). Recent investigations have shown that some ABC transporter systems in *B. subtilis* play biological roles outside the context of simply transporting molecules into or out of the cell (Rismondo and Schulz, 2021).

Despite the widespread presence of ABC transporter systems across the *B. subtilis* genome, relatively few high-resolution structures of these membrane protein complexes have been experimentally determined. Although cryo-electron microscopy (cryo-EM) has recently ushered in an abundance of ABC transporter structures from various organisms, structures of only three different intact ABC transporter complexes from *B. subtilis* have been deposited in the protein databank (Chaptal et al., 2022; Thaker et al., 2022; George et al., 2022). While cryo-EM has revolutionized structural biology of ABC transporters which are notoriously difficult to crystallize, state-of-the-art computational tools for protein structure prediction such as AlphaFold are also presenting new opportunities to gain insight into the three-dimensional architecture of putative ABC transporters (Varadi et al., 2023; Evans et al., 2022). In recent investigations we compared the cryo-EM structure of the *B. subtilis* ABC transporter complex BceAB with the structure predicted by AlphaFold, which revealed an impressively accurate prediction of the complex (George and Orlando, 2023; George et al., 2024).

Based on the initial success observed in predicting the overall structure of the BceAB complex, as well as the success observed by others across the field of structural biology (Partipilo and Jan Slotboom, 2024; Ilcu et al., 2023; Tordai et al., 2022), we sought to leverage the predictive power of AlphaFold to gain structural insight into the more than 70 ABC transporter complexes identified in *B. subtilis*. Presented herein are the AlphaFold predicted three-dimensional structures of ABC transport systems identified across the *B. subtilis* genome. Beyond structural prediction, our investigation extends to the categorization of these transporter systems based on latest schemes for classification of ABC transporter folds. This comprehensive analysis aims to refine our understanding of structural and functional diversity inherent to ABC transporter complexes across the *B. subtilis* genome, and guide future structural efforts where accurate predictions are still lacking.

## Results

In their initial inventory and classification of ABC transporters Quentin, Y. et al. used bioinformatic tools to search the *B. subtilis* genome individually for nucleotide binding domains (NBDs), membrane spanning domains (MSDs) and solute binding proteins (SBP) that likely belong to ABC transporters (Quentin et al., 1999). In bacteria the genes encoding for the individual components of an ABC transporter system are usually organized into operons (or operons in the same genetic neighborhood). Using this information Quentin, Y. et al. further developed a classification scheme to predict the identity and stoichiometry of individual protein components that make a complete ABC transporter complex. As a first step towards 3-dimensional structure prediction of all *B. subtilis* ABC transporters we manually curated the inventory created by Quentin, Y. et al. by visually inspecting the predicted AlphaFold structures of individual protein components and proteins in the same genetic neighborhood as predicted *B. subtilis* ABC transporters (Varadi et al., 2023; Pedreira et al., 2021). In many cases this simple visual analysis provided deeper insight into potential stoichiometries and classification schemes for individual ABC transporter complexes, such as identifying ABC transporter complexes with additional regulatory domains attached to the NBD, or fusion of two NBDs into one functional polypeptide (discussed in sections below). The end result of this visual and genetic analysis is a refined classification of *B. subtilis* ABC transporter systems based on their overall predicted topology, stoichiometry of individual components, function as an importer or exporter, and presence or absence of additional domains (Tables 1–7). In dividing individual transporter systems into different classes we followed the general outline provided by Thomas et al. that describes the most recent classification scheme for ABC transporter folds (Thomas et al., 2020). In the sections below we highlight the AlphaFold predicted structures of all *B. subtilis* ABC transporter systems broken down into discrete classes.

**TABLE 1:** Importers type I without regulatory domains.

*NBD1*	*NBD2*	*MSD1*	*MSD2*	*SBP1*	*Function*	*Full stoichiometry*	*Mean pLDDT*	*Max* *pLDDT*	*Min* *pLDDT*	*pTM*
AppD	AppF	AppB	AppC	AppA	Uptake of oligopeptides	AppDFBCA	83.66	97.03	19.81	0.81
DppD	YkfD	DppB	DppC	DppE	Uptake of dipeptides	DppDBCEYkfD	82.62	97.55	19.43	0.78
OppD	OppF	OppB	OppC	OppA	Initiation of sporulation, competence development	OppDFBCA	84.48	97.55	22.03	0.79
GlnQ	GlnQ	GlnM	GlnP	GlnH	Glutamine uptake	GlnQ_2_MPH	87.27	98.31	23.28	0.82
TcyN	TcyN	TcyL	TcyM	TcyK	Cystine uptake	TcyN_2_LMK	84.39	97.64	20.67	0.81
TcyC	TcyC	TcyB	TcyB	TcyA	Cystine and diaminopimelate uptake	TcyC_2_B_2_A	85.44	98.39	22.46	0.81
0TcyN	TcyN	TcyL	TcyM	TcyJ	Cystine uptake	TcyN_2_LMJ	83.81	97.67	19.9	0.81
YckI	YckI	YckA	YckA	YckB	Unknown	YckI_2_A_2_B	85.63	98.68	19.36	0.80
YxeO	YxeO	YxeN	YxeN	YxeM	Uptake and utilization of S-(2-succino)cysteine	YxeO_2_N_2_M	86.06	98.23	21.73	0.79
YgaL	YgaL	YgaM	YgaM	YgaA	Sulfonate uptake	YgaL_2_M_2_A	80.41	97.71	18.25	0.78
YtlC	YtlC	YtlD	YtlD	YtlA	Unknown	YtlC_2_D_2_A	84.21	97.57	26.08	0.79
PstBA	PstBB	PstC	PstA	PstS	High-affinity phosphate uptake	PstBABBCAS	82.87	98.05	22.28	0.82
ArtR	ArtR	ArtQ	ArtQ	ArtP	Arginine uptake	ArtR_2_Q_2_P	87.72	98.5	30.96	0.82
0										

**TABLE 2:** Importers type I with regulatory domains.

*NBD1*	*NBD2*	*MSD1*	*MSD2*	*SBP1*	*Function*	*Full Stoichiometry*	*Mean pLDDT*	*Max* *pLDDT*	*Min* *pLDDT*	*pTM*
MetN	MetN	MetP	MetP	MetQ	Methionine uptake	MetN_2_P_2_Q	85.71	98.63	25.88	0.83
FrlP	FrlP	FrlM	FrlN	FrlO	Uptake of sugar amines	FrlP_2_MNO	84.08	97.9	17.98	0.81
MsmX	MsmX	AraQ	AraP	AraN	Uptake of α-1,5-arabinooligosaccharides	MsmX_2_AraQPN	84.59	97.7	21.24	0.80
MsmX	MsmX	LplC	LplB	LplA	Uptake of maltodextrin, melibiose (probably), and galactooligosaccharides	MsmX_2_LplCBA	81.68	97.31	17.67	0.76
MsmX	MsmX	YtcP	YteP	YtcQ	Uptake of polygalacturonan and rhamnogalacturonan	MsmX_2_YtcQPYteP	82.11	97.55	21.65	0.76
MsmX	MsmX	AmyC	AmyD	MsmE	Uptake of melibiose and raffinose	MsmX_2_EAmyCD	85.16	97.85	21.23	0.82
MsmX	MsmX	YvdI	YvdH	YvdG	Uptake of maltodextrin, melibiose (probably), and galactooligosaccharides	MsmX_2_YvdIHG	83.82	97.35	20.73	0.79
MsmX	MsmX	YvfM	YvfL	YvfK	Uptake of maltodextrin, melibiose (probably), and galactooligosaccharides/	MsmX_2_YvfXMLK	83.84	97.33	20.52	0.79
MsmX	MsmX	RhiF	RhiG	RhiL	Uptake of rhamnose oligopeptides	MsmX_2_RhiFGL	82.94	97.92	18.13	0.78
OpuAA	OpuAA	OpuAB	OpuAB	OpuAC	Compatible solute transport	OpuAA_2_AB_2_AC	83.05	97.52	17.08	0.78
OpuBA	OpuBA	OpuBB	OpuBD	OpuBC	Compatible solute transport	OpuBA_2_BBBDBC	80.56	96.48	18.42	0.79
OpuCA	OpuCA	OpuCB	OpuCD	OpuCC	Compatible solute transport	OpuCA_2_CBCDCC	81.32	96.87	20.18	0.73

**TABLE 3:** Importers type II.

*NBD1*	*NBD2*	*MSD1*	*MSD2*	*SBP1*	*Function*	*Full Stoichiometry*	*Mean pLDDT*	*Max pLDDT*	*Min pLDDT*	*pTM*
FpbP	FpbP	FpbN	FpbO	FpbQ	Acquisition of iron	FpbP_2_NOQ	83.08	98.03	20.21	0.82
RbsA		RbsC	RbsC	RbsB	Ribose uptake	RbsAC_2_B	86.46	98.8	30.04	0.88
NupO		NupP	NupQ	NupN	Uptake of guanosine	NupNOPQ	85.27	98.68	18.52	0.84
FhuC	FhuC	FhuB	FhuG	YxeB	Siderophore uptake	FhuC_2_BGYxeB	80.73	97.75	14.84	0.79
FhuC	FhuC	FhuB	FhuG	FhuD	Siderophore uptake	FhuC_2_BGD	80.49	97.7	14.54	0.80
YvrA	YvrA	YvrB	YvrB	YvrC	Uptake of cobalamin	YvrA_2_B_2_C	82.94	98.37	18.44	0.80
FedF	FedF	FedD	FedE	FedC	Iron uptake	FedF_2_DEC	82.64	98.12	20.27	0.82
YusV	YusV	YfiZ	YfhA	YfiY	Acquisition of iron	YusV_2_YfiYZYfhA	84.42	98.19	20.46	0.84
YusV	YusV	FeuB	FeuC	FeuA	Acquisition of iron	YusV_2_FeuBCA	81.86	97.85	16.97	0.80
MntB	MntB	MntC	MntD	MntA	Manganese uptake	MntB_2_CDA	81.70	98.27	20.33	0.84
ZnuC	ZnuC	ZnuB	ZnuB	ZnuA	Zinc uptake	ZnuC_2_B_2_A	79.49	97.66	20.49	0.82
FecF	FecF	FecD	FecE	FecC	Acquisition of iron / citrate	FecF_2_DEC	82.64	98.12	20.27	0.82
FecF	FecF	FecD	FecE	YhfQ	Acquisition of iron / citrate	FecF_2_DEYhfQ	81.82	98.12	21.69	0.82

**TABLE 4:** Exporters type IV.

*NBD1*	*NBD2*	*MSD1*	*MSD2*	*Function*	*Full Stoichiometry*	*Mean pLDDT*	*Max pLDDT*	*Min pLDDT*	*pTM*
CydD		CydC		Required for expression of cytochrome bd	CydDC	86.93	98.1	38.21	0.8
YfiB		YfiC		Unknown	YfiBC	88.64	98.06	40.56	0.84
YknU		YknV		Unknown	YknUV	88.25	98.2	27.43	0.83
BmrC		BmrD		Multiple antibiotic resistance	BmrCD	87.29	97.73	28.52	0.81
YwjA		YwjA		Unknown	YwjAA	87.47	98.64	30.42	0.86
YgaD		YgaD		Unknown	YgaDD	88.41	98.18	36.37	0.81
BmrA		BmrA		Multiple antibiotic resistance	BmrAA	84.92	98.03	21.77	0.8
SunT		SunT		Sublancin export and processing	SunTT	80.69	96.86	21.41	0.77

**TABLE 5:** Exporters type V.

*NBD1*	*NBD2*	*MSD1*	*MSD2*	*Function*	*Full Stoichiometry*	*Mean pLDDT*	*Max pLDDT*	*Min pLDDT*	*pTM*
TagG	TagG	TagH	TagH	Biosynthesis of teichoic acid	TagG_2_H_2_	78.97	97.89	19.18	0.69
YcbN	YcbN	YcbO	YcbO	Unknown	YcbN_2_O_2_	84.02	98.38	29.84	0.81
AlbC	AlbC	AlbD		Export of antilisterial bacteriocin (subtilosin)	AlbC_2_D	88.17	98.68	37.57	0.85
EcsA	EcsA	EcsB		Regulation of the secretion apparatus and intra-membrane proteolysis	EcsA_2_B	90.29	98.37	32.62	0.86
YthP	YthP	YthQ		Unknown	YthP_2_Q	88.81	98.49	36.81	0.85
YhcG	YhcG	YhcE	YhcE	Unknown	YhcG_2_E_2_	90.84	98.51	57.08	0.84
YybJ	YybJ	YybK	YybL	Unknown	YybJ_2_KL	84.03	98.21	23.93	0.7
YdbJ	YdbJ	YdbK	YdbK	Unknown	YdbJ_2_K_2_	85.85	98.33	27.19	0.83
YtrB	YtrB	YtrC	YtrD	Unknown	YtrB_2_CD	87.65	98.57	34.09	0.84
YvfR	YvfR	YvfS	YvfS	Unknown	YvfR_2_S_2_	86.11	98.13	36.47	0.81
YxlF	YxlF	YxlG	YxlG	Unknown	YxlF_2_G_2_	84.60	97.47	40.31	0.78
SkfE	SkfE	SkfF		Export of the spore killing factor	SkfE_2_F	85.37	97.63	35.93	0.8

**TABLE 6:** Exporters type VII.

*NBD1*	*NBD2*	*MSD1*	*MSD2*	*Function*	*Full Stoichiometry*	*Mean pLDDT*	*Max pLDDT*	*Min pLDDT*	*pTM*
FtsE	FtsE	FtsX	FtsX	Control of cell wall synthesis	FtsE_2_X_2_	84.24	98.32	29.57	0.73
YclH	YclH	YclI	YclI	Unknown	YclH_2_I_2_	76.76	97.44	17.67	0.67
YknY	YknY	YknZ	YknZ	Resistance to SdpC toxin	YknY_2_Z_2_	81.74	98.1	27.82	0.65
YtrE	YtrE	YtrF	YtrF	Unknown	YtrE_2_F_2_	83.96	98.35	35.31	0.76
YvrO	YvrO	YvrN	YvrN	Unknown	YvrO_2_N_2_	81.66	98.04	27.0	0.69
BceA	BceA	BceB		Protection of cell wall biosynthetic targets from inhibition by antimicrobial peptides	BceA_2_B	86.63	97.88	31.57	0.81
PsdA	PsdA	PsdB		Protection of cell wall biosynthetic targets from inhibition by antimicrobial peptides	PsdA_2_B	85.30	98.01	25.7	0.78
YxdL	YxdL	YxdM		Protection of cell wall biosynthetic targets from inhibition by antimicrobial peptides	YxdL_2_M	85.41	97.73	24.0	0.78

**TABLE 7:** Exporters hybrid type V/VI and IV/V.

*NBD1*	*NBD2*	*MSD1*	*MSD2*	*Function*	*Full Stoichiometry*	*Mean pLDDT*	*Max pLDDT*	*Min pLDDT*	*pTM*
YhcH	YhcH	YhcI	YhcI	Unknown	YhcH_2_I_2_	81.56	97.51	26.44	0.8
YhaQ	YhaQ	YhaP	YhaP	Unknown	YhaQ_2_P_2_	83.15	97.99	36.21	0.81
LnrL	LnrL	LnrM	LnrN	resistance to linearmycin	LnrL_2_MN	82.11	98.05	38.55	0.78
NatA	NatA	NatB	NatB	sodium export	NatA_2_B_2_	86.23	97.72	37.42	0.84
YjkB	YjkB	YjkA	YjkA	Unknown	YjkB_2_A_2_	87.44	98.13	23.23	0.85

### Importers and regulatory domains

**FIGURE 1:**
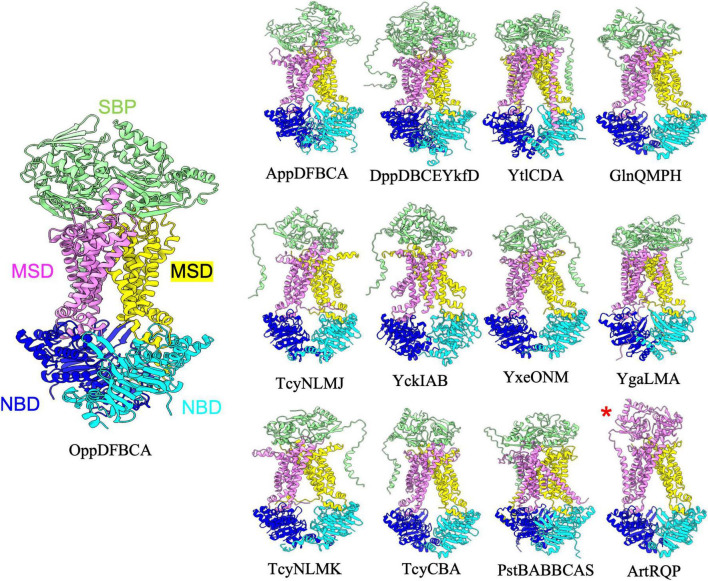
Importers type I without regulatory domain. Shown above are cartoon representations highlighting the predicted structure of Type I ABC importers that do not contain regulatory domains on the NBDs. One NBD is shown in blue and the other is shown in cyan. The violet above the blue NBD represents one of the MSD proteins while the yellow above the cyan NBD represents the other MSD protein. The light green domain on the top of the structure is the SBP. ArtRQP has a unique topology in which the SBP is fused into one polypeptide with one of the MSDs (shown with a red asterisk).

ABC transporters that mediate uptake of small molecules and other compounds from the extracellular environment (importers) are generally categorized into three different types based on their overall transmembrane domain (TMD) architecture (Thomas et al., 2020). Type I importers adopt a fold similar to the maltose transporter system MalFGK_2_E of *E. coli* (Oldham and Chen, 2011), whereas Type II importers resemble the vitamin B12 uptake system BtuC_2_D_2_F from *E. coli* (Hvorup et al., 2007). Type III importers are also known as the ECF transporters, and have a vastly different TMD fold and overall mechanism than other classical ABC transporter systems, and are not considered here in our current analysis of *B. subtilis* ABC transporters. Type I and II importers both utilize a periplasmic solute binding protein (SBP) to bind and transfer substrates to the TMD of the ABC transporter for further import (Figure 1).

Some ABC transporters have an extra regulatory domain that extends off of the NBD, and often plays a significant role in modulating the overall conformation and function of the complex. Examples of such regulatory domains include those in MalK of the *E. coli* maltose transporter system which bind the enzyme IIA*^glc^* and the transcriptional activator MalT (Boos and Shuman, 1998; Chen et al., 2003), and those in Wzt of the WzmWzt O-antigen transporter of *Aquifex aeolicus* which recognize O-antigen modifications (Bi and Zimmer, 2020). In the analysis below we divide predicted *B. subtilis* ABC importer structures based on classification into Type I or II, and whether or not they possess regulatory domains on the NBDs.

### Type I Importers without regulatory domains

The *B. subtilis* genome contains thirteen type I ABC importers that lack a regulatory domain on the NBD (Figure 1 and Table 1). For all such transporters, AlphaFold predicted the periplasmic SBP interacting with the top of the MSDs such that the small molecule binding cleft of the SBP is oriented to facilitate transfer of transported substrates to the MSDs. Such a configuration is highly reminiscent of the structure of *E. coli* maltose transporter in a pre-translocation intermediate state (Oldham and Chen, 2011). Although the type I importers adopt overall similar folds, slight structural diversity still exists within the configuration of individual complexes. For instance, comparing the prediction for YtlCDA and GlnQMPH (top row, Figure 1) reveals a domain swap configuration for one transmembrane helix of YtlCDA and no such domain swapping in the TMDs of GlnQMPH. Moreover, the prediction for the ArtRQP complex (bottom row, Figure 1) demonstrates that this transporter is unique among type I importers because the SBP is fused to one of the MSD transmembrane helices. Overall, the AlphaFold predictions of Type I transporters reveal a similar architecture with slight deviations between complexes that are involved in uptake of substances including amino acids, di- and oligopeptides, sulfonate, and phosphate (Table 1). The predictions presented here suggest that the overall structure of type I importers is conserved across similar complexes in the *B. subtilis* genome, and the ability of each individual ABC transporter to recognize and transport specific solutes is likely dependent on the specific amino acid composition of substrate binding sites in the SBP and TMDs, rather than gross differences in transporter structure.

### Type I importers with regulatory domains

**FIGURE 2:**
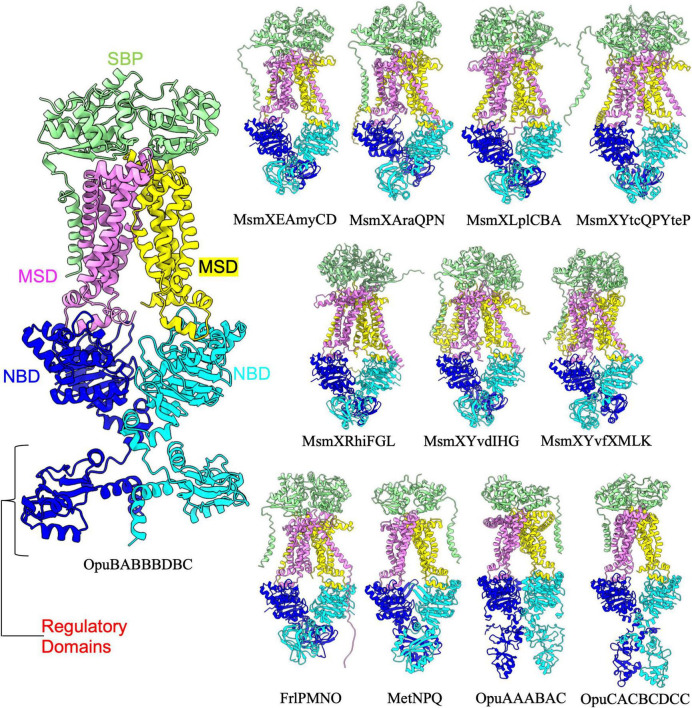
Importers type I with regulatory domain. Shown above are cartoon representations highlighting the predicted the structure of Type I ABC importers that contain a regulatory domain fused to the NBD. One NBD is show in blue and the other is shown in cyan. The violet above the blue NBD represents one of the MSD proteins while the yellow above the cyan NBD represents the other MSD protein. The light green domain on the top of the structure is the SBP. The regulatory domains are highlighted in the large representation of OpuBABBBDBC for clarity.

Among type 1 importers there are an additional eleven ABC transporter complexes in which the NBDs contain a regulatory domain (Figure 2 and Table 2). Interestingly, it has been proposed that some NBDs can act as “multitask” ATPases to energize multiple different ABC transporters. One of the best examples of such an NBD is MsmX, which has previously been shown to power at least 6 different ABC transporter systems in *B. subtilis* (Leisico et al., 2020). AlphaFold was able to reliably predict (based on mean pLDDT score) complexes for these 6 transporter complexes each containing MsmX as an NBD (Figure 2). The regulatory domain extending off of MsmX and FrlP adopts a transporter-associated OB (TOBE) domain fold similar to that of Malk in the *E. coli* maltose transporter complex, which acts as a hub to recruit regulatory proteins (Boos and Shuman, 1998; Chen et al., 2003). In contrast to the TOBE domain observed in MsmX, the regulatory domain of MetN in the MetNPQ complex adopts a NIL domain fold (PFAM: PF09383) that is commonly found in the NBD of many ABC transporters. On the other hand, the regulatory domains found in OpuAA, OpuBA, and OpuCA are cystathionine beta-synthase (CBS) domains (PFAM: PF00571), which have been shown to act as small molecule binding molecular switches (Baykov et al., 2011), and likely play a regulatory role in the conformational switching and overall function of these ABC transporters. Aside from the additional regulatory domains extending off of the NBD, all twenty-four type I importers in *B. subtilis* were predicted by AlphaFold to adopt a highly similar overall architecture reminiscent of the maltose transporter.

### Type II importers

**FIGURE 3:**
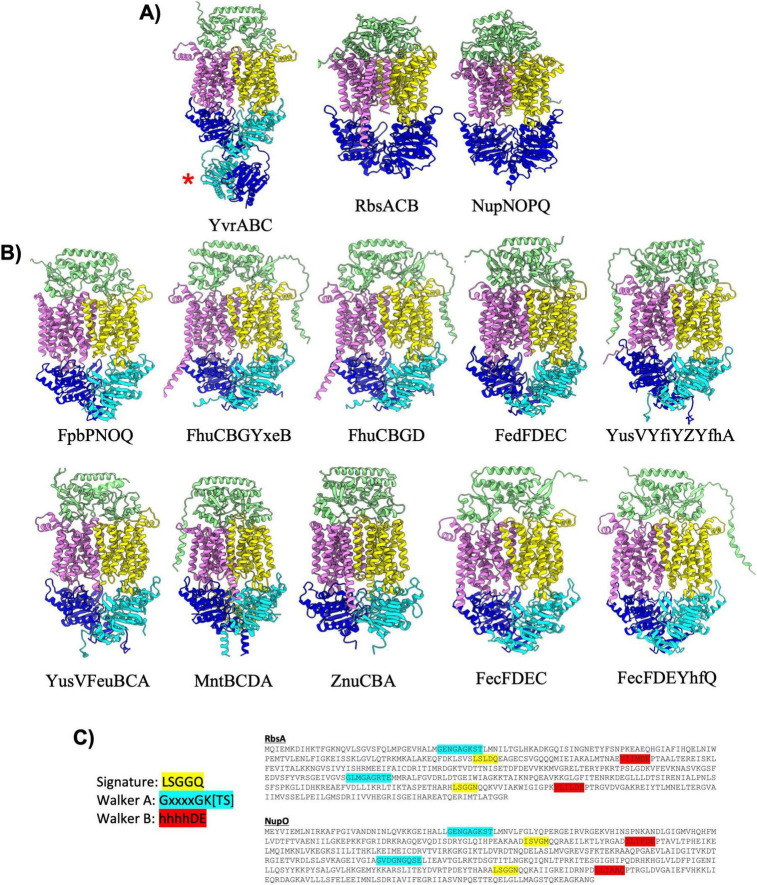
Importers type II. **(A)** Shown are cartoon representations highlighting the predicted structure of Type II ABC importers with regulatory domains or fused NBDs. One NBD is shown in blue and the other is shown in cyan. The violet above the blue NBD represents one of the MSD proteins while the yellow above the cyan NBD represents the other MSD protein. The light green domain on the top of the structure is the SBP. YvrABC contains a regulatory domain fused to the NBD (red asterisk). Both RbsACB and NupNOPQ have a unique topology in which the NBDs are fused into one polypeptide shown in the large blue domain. **(B)** Cartoon representations of type II importers without regulatory domains. Coloring is the same as in **(A)**. **(C)** Amino-acid sequences of the RbsA and NupO fused NBD proteins. Key ATPase motifs are highlighted, demonstrating one degenerate ATPase site.

*B. subtilis* contains twelve type II importers that resemble the classical transporter BtuC_2_D_2_ with 20 TM helices and a SBP docking on top of the extracellular region of the MSDs (Figure 3 and Table 3; Hvorup et al., 2007; Locher et al., 2002). AlphaFold predicted a similar overall configuration for each of the type II importer complexes with NBDs separated in a nucleotide free conformation, and the SBP interacting with the top extracellular region of the MSDs. Among the twelve type II importers, the YvrABC complex is unique in that it is the only complex with regulatory domains extending off the NBDs into the cytosol (Figure 3). Like the *E. coli* transporter BtuC_2_D_2_, YvrABC is proposed to mediate uptake of vitamin B12 (cobalamin) in *B. subtilis* (Table 3). The regulatory domain of the YvrA subunit adopts what appears to be an FTH/DUF38 domain fold (PFAM: PF01827), which is presumed to act as a potential protein-protein interaction module. The presence of these regulatory domains in YvrABC suggests additional layers of regulating vitamin B12 import that may not be present in similar systems such as BtuC_2_D_2_ in *E. coli*.

Among all of the predicted *B. subtilis* ABC transporter complexes (both importers and exporters), the RbsACB and NupNOPQ type II importer complexes are unusual in that the two NBDs are fused together into a single polypeptide (Figure 3, red asterisk). Fusion of NBDs into a single polypeptide is relatively rare among prokaryotic ABC transporters, and it is currently unclear what if any role such a fusion may play in regulating transporter dynamics and function. It is interesting to note that sequence analysis of the RbsA and NupO NBDs suggests that one of the ATP binding sites in each of these proteins is degenerate (not competent for ATP binding/hydrolysis) as both proteins contain at least one ABC signature motif sequence that deviates significantly from the canonical LSGGQ sequence, in both proteins the second Walker A motif does not contain a lysine residue critical for coordinating bound ATP, and in the second Walker B motif of NupO the critical aspartic acid and glutamate residues are not present (Figure 3B). While degenerate ATP binding sites are not unusual in ABC transporters (Stockner et al., 2020), it is interesting that the only two NBDs that are fused into a single polypeptide in *B. subtilis* contain such a degenerate ATPase sequence. This observation raises intriguing questions about the evolutionary significance and potential functional implications of these degenerate ATP binding sites in the context of a fused NBD, which likely arose through gene duplication and eventual fusion. Further investigation is warranted to provide insight into how this unique structural feature may influence transporter activity and regulation.

### Type IV exporters

**FIGURE 4:**
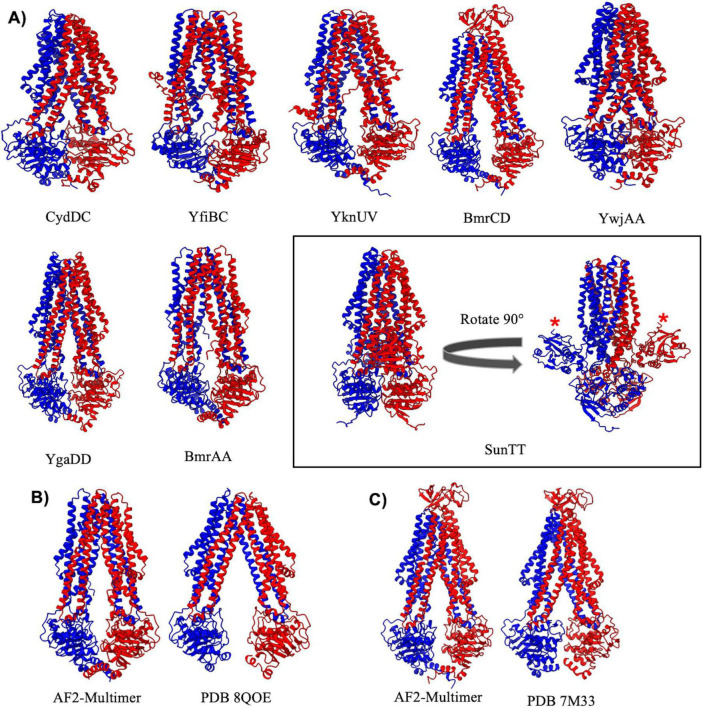
Importers type IV. **(A)** Shown above are cartoon representations highlighting the predicted structure of Type IV ABC exporters. YwjA, YgaD, BmrA, and SunT are homodimers of the same protein, whereas all other type IV exporters exist as heterodimers of two individual proteins. The SunTT protein has a unique architecture with an additional peptidase domain fused to the N-terminus (shown with a red asterisk). **(B,C)** Comparisons of AlphaFold-2 Multimer predictions and experimentally determined structures for BmrA **(B)** and BmrCD **(C)**.

ABC transporters belonging to the type IV exporter class adopt a fold similar to the founding member Sav1886 from *Staphylococcus aureus* (Dawson and Locher, 2006), and display 12 long transmembrane helices that extend well into the cytosol where they engage the NBDs (Figure 4). Type IV exporters also invariably display a domain swapped configuration, with transmembrane helix 4 and 5 from one MSD interacting through the intervening coupling helix with the NBD from the opposite half of the transporter. Two out of the three *B. subtilis* ABC transporters that have had experimental structures determined (BmrA and BmrCD) belong to the type IV exporter class (Chaptal et al., 2022; Thaker et al., 2022; Tang et al., 2023). Experimental structures of BmrA have been determined in an inward facing nucleotide free conformation (Di Cesare et al., 2024), and ATP bound conformations with both NBDs dimerized around bound ATP molecules (Chaptal et al., 2022). The AlphaFold-2 Multimer prediction of BmrA more closely resembles the inward facing nucleotide free cryo-EM structure of the complex (Di Cesare et al., 2024), although a significant difference in the degree of opening between the NBDs is observed when comparing the predicted and experimentally determined structure (Figure 4B). This difference in conformation could potentially be attributed to the solubilization condition (detergent, nanodisc, amphipol, etc…) used in experimental structure determination procedures, which has been shown to heavily influence the conformation of ABC transporters (Hoffmann et al., 2024). In contrast, the AlphaFold-2 Multimer prediction of BmrCD is virtually superimposable with the cryo-EM structure of BmrCD in an inward facing conformation (Thaker et al., 2022; Figure 4C). AlphaFold-2 Multimer predicted the structure of all other type IV exporters to be in an inward facing nucleotide free conformation, except for the YwJAA complex that displays collapsed NBDs in a conformation that would be expected upon binding of ATP.

Among type IV importers the BmrCD and SunTT complexes stand out with distinct structural features. BmrCD contains an extracellular domain that adopts a Penicillin Binding Protein 4 C-terminal domain fold of currently unknown function (Thaker et al., 2022). On the other hand, the SunTT complex contains an N-terminal C39 peptidase domain and an overall configuration nearly identical to that observed for the “PCAT” transporter from *C. thermocellum* (Lin et al., 2015). The C39 peptidase domain of SunT cleaves the pro-peptide of the glycocin antibiotic sublancin to generate the mature antimicrobial peptide, which is then transported out of the cell by SunT (Håvarstein et al., 1995). Aside from BmrCD and SunTT all other AlphaFold predictions of *B. subtilis* type IV transporters are highly similar to classical ABC transporters such as Sav1886 or MsbA (Dawson and Locher, 2006; Mi et al., 2017). These transporters all display domain swapping of transmembrane helixes 4 and 5 across the dimer interface, and long transmembrane helices that extend well into the cytosol with coupling helices that engage the NBDs at an extended distance from the plane of the lipid membrane compared to other ABC importers and exporters.

### Type V exporters

**FIGURE 5:**
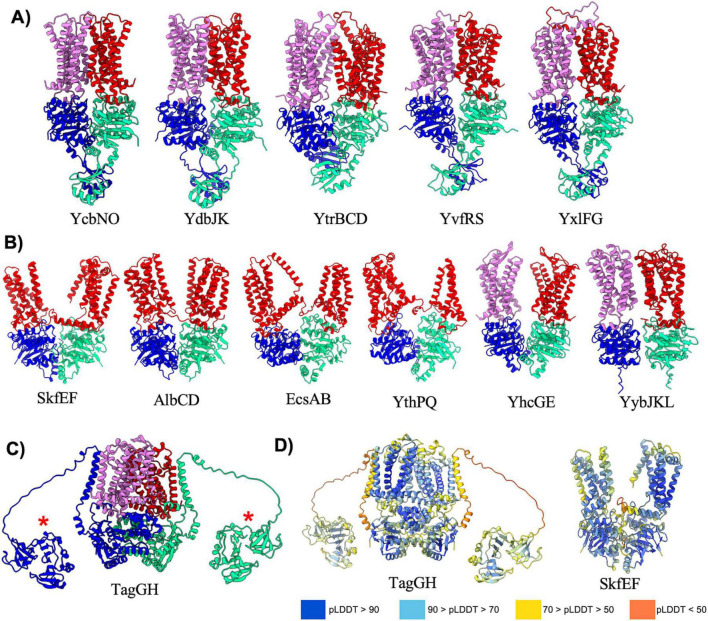
Exporters type V. Shown above are cartoon representations highlighting the predicted structure of Type V ABC exporters. One NBD is shown in blue and the other is shown in light green. The violet above the blue NBD represents one of the MSD proteins while the red above the light green NBD represents the other MSD protein. **(A)** Predicted structures of Type V exporters that contain a regulatory domain fused to the NBD. **(B)** Predicted structures of Type V exporters that do not contain a regulatory domain fused to the NBD. AlbCD, EcsAB, and YthPQ have a unique topology in which the two MSDs are fused together into a single polypeptide shown in red. **(C)** TagGH is an unusual type V exporter in which the NBDs are fused to a transmembrane helix that extends into an extracellular domain (red asterisk). Although AlphaFold predicts the extracellular domain to be in the cytosol near the NBDs, in *B. subtilis* this domain would extend into the extracellular space towards the peptidoglycan layer. **(D)** Structures of various type V exporters colored by pLDDT demonstrating poor pLDDT scores in flexible linker regions.

Type V exporters (Table 5) are characterized by their distinct fold of the TMD and a lack of domain swapping in the transmembrane helices. Transporters that adopt the type V exporter fold are typified by the Wzm family of transporters in bacteria (Spellmon et al., 2022), and the ABCG (Taylor et al., 2017; Lee et al., 2016) and ABCA (Plummer-Medeiros et al., 2023) families in humans. *B. subtilis* contains 11 type V exporters that can be further divided into two classes, those with regulatory domains (5 transporters) or without regulatory domains (6 transporters) extending off of the NBD (Figure 5). Interestingly, AlphaFold predicted reasonable ABC transporter complex structures for all type V exporters with regulatory domains (Figure 5A), but generally struggled to provide accurate predictions for those without regulatory domains (Figure 5B). The regulatory domains on all type V exporters adopt a similar fold to that seen previously in the human ABCA1 (Plummer-Medeiros et al., 2023) and ABCA4 (Scortecci et al., 2021; Xie et al., 2021) structures with two alpha-helices and a 4-stranded anti-parallel beta-sheet (Figure 5A). Among type V exporters without regulatory domains the SkfEF, AlbCD, EcsAB, and YthPQ complexes are unique in that all of the TMD is encoded by a single polypeptide versus two individual polypeptides (Figure 5B). AlphaFold appears to struggle predicting an accurate structure for these complexes as evidenced by the large separation in the TMD region that is likely not representative of the true structure. Although these predictions can display a lower pLDDT score in the linker region connecting the TMD two helical bundles (Figure 5D), the mean pLDDT score and pTM score for most of these predictions are similar to other well predicted complexes. Thus, assessing the quality of a given prediction based solely on metrics such as pLDDT and pTM may fail to capture possible inaccuracies. Similarly, in the YhcGE and YybJKL complexes which contain two separate polypeptides for each individual TMD, AlphaFold appears to correctly model the fold and interaction of the TMD and NBD, but there is a large gap between the individual TMDs that seems unlikely to be accurate.

The final member of the type V exporters in *B. subtilis* is the TagGH complex that is involved in transport of teichoic acids (Lazarevic and Karamata, 1995). The AlphaFold predicted structure of TagGH reveals a similar overall fold as the TarGH teichoic acid transporter from *Alicyclobacillus herbarius* (Chen et al., 2020), and predicts the complex to be in a nucleotide bound conformation similar to that observed in the TarGH cryo-EM structure. However, TagGH is unique in that the TagG NBD subunit contains a transmembrane domain that positions the c-terminus of the protein in the extracellular region (Figure 5C). The C-terminus of TagG is predicted as a well folded domain that has some homology to SH3b domains known to bind polymers such as teichoic acid in the cell-wall (Shen et al., 2020). A long (∼30 residues) and likely flexible linker connects the transmembrane helix and C-terminal domain of TagG, and AlphaFold places the C-terminal domain in the cytoplasm near the NBDs (Figure 5C). This is an incorrect positioning as the C-terminal domain will certainly reside on the extracellular side of the plasma membrane, and likely extends up from the plane of the bilayer towards the thick peptidoglycan layer surrounding *B. subtilis*. The incorrect positioning of this domain is likely a consequence of AlphaFold not having knowledge of the membrane location, and the low pLDDT score in the linker connecting the TagG NBD and C-terminal extension (Figure 5D). Nevertheless, the AlphaFold prediction provides first insight into the fold and plausible function of the TagG C-terminal domain.

### Type VII exporters

**FIGURE 6:**
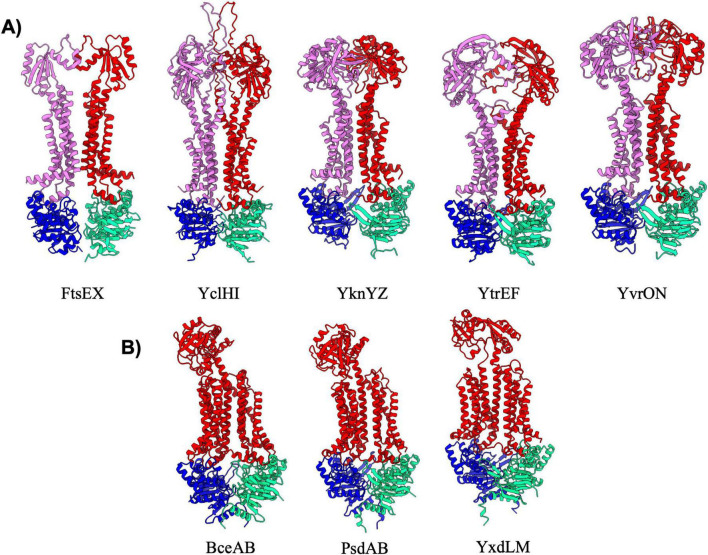
Exporters type VII. Shown above are cartoon representations highlighting the predicted structure of Type VII ABC exporters. **(A)** Predictions of type VII exporters that are similar to MacB. One NBD is shown in blue and the other is shown in light green. The violet above the blue NBD represents one of the MSD proteins while the red above the light green NBD represents the other MSD protein. **(B)** Type VII transporters that adopt a Bce-like fold. These three transporters have a unique topology in which a single MSD contains a singular extracellular domain.

Type VII exporters are also known as “mechanotransducers” as these ABC transporters do not necessarily move a substance across the membrane, but rather act to activate extracellular enzymes (Hao et al., 2024), extract lipoproteins from the membrane (Sharma et al., 2021), or pump small molecules through conduits to the extracellular space (Crow et al., 2017). The type VII exporter fold is typified by the structure of the MacB multidrug transporter from *E. coli* (Crow et al., 2017), and all type VII exporters have been found to have FtsX-like folds within the TMD architecture. In *B. subtilis* there are eight type VII exporters that can be divided into two distinct classes. The first class contains the FtsEX, YclHI, YknYZ, YtrEF, and YvrON complexes, all of which adopt a classical MacB type fold. These transporters are overall two-fold symmetrical, and contain two MSDs that each adopt FtsX-like 4-TM helical bundles for a total of eight transmembrane helices, as well as a large extracellular domain of diverse size and structure (Figure 6A). Interestingly, the type VII transporters appear to have some of the lowest pTM scores across all transporters in *B. subtilis*. However, we believe these below average scores are likely a consequence of poor confidence (pLDDT) in several dynamic loop regions and the greasy interaction between FtsX like folds of the two MSDs, rather than any major overall inaccuracy in the structural prediction. Aside from FtsEX which is known to activate a cell-wall hydrolase during cell division, the function of other type VII exporters within this category remains relatively obscure or ill-defined (Table 6).

The second category of type VII exporters is the “Bce-type” transporters which are named after the founding member BceAB from *B. subtilis* (George et al., 2022). There are three Bce-type transporters in *B. subtilis* (Figure 6B) that all adopt an overall configuration that is similar to that observed in the cryo-EM structure of nucleotide-free BceAB (George et al., 2022). Bce-type transporters are distinct from other type VII transporters in that they contain ten transmembrane helices encoded in a single polypeptide, contain only a single extracellular domain, and lack overall symmetry in the complex. The largest difference between the three Bce-type transporters in *B. subtilis* lies in the fold of the extracellular domain, which is proposed to mediate recognition of highly diverse antimicrobial peptides by each of the complexes (George et al., 2024). Bce-type transporters are known to form a membrane protein complex with a histidine kinase, which together work in tandem to sense antimicrobial peptides and initiate signaling to mediate resistance (George and Orlando, 2023; George et al., 2024).

### Hybrid exporters

**FIGURE 7:**
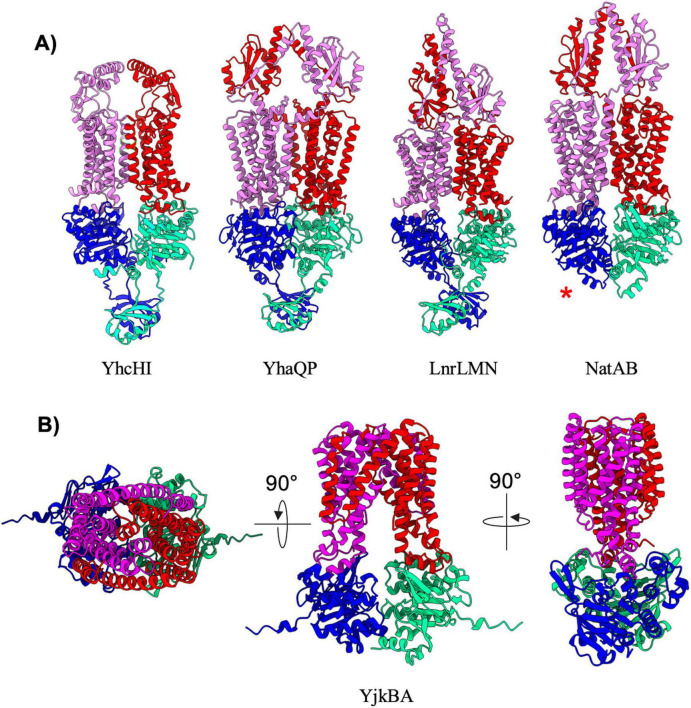
Hybrid exporters. **(A)** Shown are cartoon representations highlighting the predicted structure of Type V/VI hybrid ABC exporters. One NBD is shown in blue and the other is shown in light green. The violet above the blue NBD represents one of the MSD proteins while the red above the light green NBD represents the other MSD protein. NatAB is the only protein in this set that does not contain a regulatory domain fused to its NBDs (shown with a red asterisk). **(B)** Shown are cartoon representations highlighting the predicted structure of the Type IV/V hybrid ABC exporter YjkBA. One NBD is shown in blue and the other is shown in cyan. The violet above the blue NBD represents one of the MSD proteins while the red above the light green NBD represents the other MSD protein.

The remaining five exporters in *B. subtilis* (Table 7; YhcHI, YhaQP, LnrLMN, NatAB, and YjkBA) display AlphaFold predicted structures that appear to be a hybrid between different classes of transporters. The YhcHI, YhaQP, LnrLMN, and NatAB complexes display a similar TMD architecture as type V exporters with a short stature and lack of domain swapping in the TMD region, but also display relatively large extracellular domains (Figure 7A). The extracellular domains of these transporters are similar in size and location to the beta jelly-roll domains found in the type VI transporter complex LptB_2_FG from Gram-negative (Li et al., 2019; Owens et al., 2019). For this reason, we have designated YhcHI, YhaQP, LnrLMN, and NatAB *B. subtilis* transporters as hybrids between type V and type VI transporters. YhaQP, LnrLMN, and NatAB all display domain swapped extracellular domains that adopt a similar fold of a five-stranded beta-sheet with flanking alpha-helices (Figure 7A). Searches for similar domains or protein folds for these extracellular domains revealed no known protein domains, suggesting a novel overall fold. In contrast to YhaQP, LnrLMN, and NatAB, the YhcHI complex displays a smaller extracellular domain composed of two alpha-helices that form a coiled-coil. Interestingly, among the transporters classified as type V/VI hybrids, the only complex that lacks regulatory domains on the NBD is NatAB (Figure 7A).

The final ABC transporter complex in *B. subtilis* is the YjkBA complex, which has an unusual topology unlike any of the other transporters found in this organism (Figure 7B). The YjkBA complex displays a total of fourteen transmembrane helices that are arranged in a domain swapped configuration similar to that seen in type IV transporters (Figure 4). However, unlike type IV transporters the transmembrane helices of the YjkA do not extend far below the plane of the lipid bilayer like type IV transporters, and as a result the YjkB NBDs are located closer to the lipid bilayer than they are in type IV transporters. Moreover, YjkA lacks an N-terminal “elbow helix” that is typical of type IV transporters (Thomas et al., 2020). The short stature (location of the NBDs close to the membrane) and compact nature of the TMD in the YjkBA complex is somewhat reminiscent of type V transporters. For these reasons we have designated YjkBA as a hybrid type IV/V exporter, which adopts an overall architecture unlike any ABC transporter of known structure.

### Comparison of AlphaFold-3 predictions with ATP and experimentally determined structures

**FIGURE 8:**
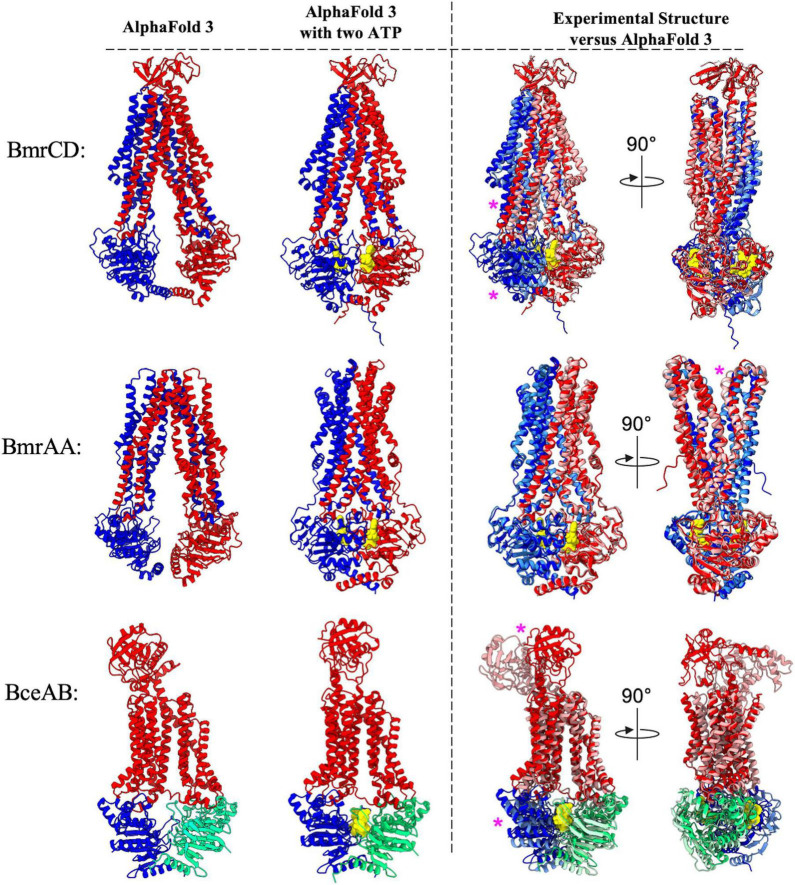
Comparison of AlphaFold 3 predictions with ATP versus experimental structures. Shown above are cartoon representations highlighting the predicted structure of the ABC transporters that have had experimental structures determined. Predictions utilizing AlphaFold 3 with or without 2 ATP molecules are shown to the left of the dotted vertical black line. Individual polypeptide chains are colored blue, red, or green. ATP is shown as yellow spheres. To the right of the dotted vertical black line is an overlay showing AlphaFold 3 predicted structures (darker colors) aligned to experimentally determined structures which are shown in lighter colors (BmrCD PDB:8FHK, BmrAA PDB:7OW8, BceAB PDB:7TCH). Magenta asterisks highlight regions of discrepancy between the experimental and predicted structures.

During preparation of this manuscript the latest version of AlphaFold (AlphaFold-3) was released (Abramson et al., 2024). Although the source code for this new implementation of AlphaFold was not made available, the ability to perform predictions between proteins and a limited list of small molecules was provided in the form of AlphaFold Server.^1^ Among the list of available ligands is the nucleotide ATP, which provides an opportunity to explore the ability of AlphaFold-3 in predicting the conformational changes that are thought to be initiated by ATP binding to ABC transporters. To explore this possibility, we performed AlphaFold-3 predictions either in the absence or presence of ATP molecules for the three *B. subtilis* ABC transporters that have had experimental structures determined (George et al., 2022; Tang et al., 2023; Di Cesare et al., 2024). In the absence of ATP, the AlphaFold-3 predictions for BmrCD, BmrA, and BceAB reveal an essentially identical prediction to that which was provided by AlphaFold-2 Multimer (Figure 8, left). When two copies of an ATP molecule were included in these predictions, AlphaFold-3 placed each ATP molecule in the correct location between the Walker A and signature motif of each NBD in the ABC transporter complexes, and in all three complexes inclusion of ATP induced a conformational shift towards a nucleotide bound conformation (Figure 8, left).

Although AlphaFold-3 reliably predicted the general ATP binding sites in each complex, the conformational shifts observed in each complex show some discrepancies to experimentally determined structures. The AlphaFold-3 prediction of BmrA bound to ATP is nearly identical to the experimentally determined cryo-EM structure (PDB: 7OW8) (Di Cesare et al., 2024), with only slight discrepancies in the relative position of the loop extending between transmembrane helix 1 and 2 (Figure 8, right). On the other hand, the AlphaFold-3 prediction of BmrCD in the presence of ATP reveals significant differences in the positioning of the NBDs and coupling helices relative to the experimentally determined cryo-EM structure (PDB:8FHK) (Tang et al., 2023). In the AlphaFold-3 predicted structure of BmrCD with ATP the NBDs are not fully dimerized to sandwich ATP between each NBD, and further conformational change (toward the experimentally determined structure) would be required for BmrCD to adopt a conformation that is competent for ATP hydrolysis (Figure 8, right). Finally, the AlphaFold-3 predicted structure of BceAB with ATP molecules is strikingly different than the experimentally determined cryo-EM structure (PDB:7TCH) (George et al., 2022). In the AlphaFold-3 prediction of BceAB the NBDs are not fully dimerized in a conformation that would support ATP hydrolysis, the transmembrane helices do not collapse into a more compacted state as seen in the cryo-EM structure, and the large extracellular domain is in a completely different conformation than that observed in the cryo-EM structure. Thus, while AlphaFold-3 was able to predict conformational changes elicited by ATP binding, in two out of the three cases explored here the predicted structures of ABC transporters bound to nucleotides (ATP) deviate in several ways from the same structures determined through experimental methods. The implications of these findings are explored in further detail in the following discussion.

## Discussion

Although the simple soil bacterium *B. subtilis* contains nearly twice as many ABC transporters as complex multicellular organisms such as humans, relatively little progress has been made in experimental structure determination of ABC transporter complexes from this organism. In a similar way that cryo-EM revolutionized structural biology, artificial intelligence-based protein structure prediction algorithms such as AlphaFold are currently paving the way for another new era in structural biology. To gain structural insight into the ∼70 ABC transporters from *B. subtilis* that have not had experimental structures determined, we utilized AlphaFold to predict the 3-dimensional structures of all ABC transporter systems in this organism. The structural predictions presented here provide the first template to begin understanding the structural diversity inherent to ABC transporters in *B. subtilis*.

Our comprehensive AlphaFold predictions of all ABC transporter complexes in *B. subtilis* reveal several important points for consideration. First, in general AlphaFold does a remarkable job at predicting the overall structure of ABC transporter complexes in *B. subtilis*. The predicted structures of type I, II, IV, VII, and most type V transporters (Figures 1–6) are likely to be quite accurate, although further experimental validation is always warranted. Second, there are some instances where AlphaFold performs poorly and generates structural predictions that seem obviously flawed. Examples of such poor predictions are mostly in the type V ABC transporters (Figure 5B), where implausible predictions are readily apparent from the large gaps between transmembrane helices and unlikely configuration of the overall complexes. Although large gaps between TMD bundles have been observed previously in transporters such as human ABCA1 (Qian et al., 2017) and ABCA4 (Scortecci et al., 2021; Xie et al., 2021), these transporters are involved in lipid export from the plasma membrane and also have large extracellular domains that extend above the gap between the TMDs. None of the predictions for type V exporters presented here contain such extracellular domains, and given the often low pLDDT score for the linker connecting their wide spread TMD domains, it seems likely that these predictions do not reflect the true structure of the complex. Nevertheless, experimental validation should be performed to confirm these assumptions. While the power of AlphaFold in providing novel insight into the assembly of protein complexes is undeniable, there is certainly still room for improvement. Such improvement is likely to come in part through continued experimental structure determination of protein complexes that AlphaFold struggles to predict accurately.

Another significant observation from our analysis is the fact that AlphaFold suggests several new folds and architectures that have never been seen in ABC transporters. For instance, the type V/VII hybrid (Figure 7) and type IV/V hybrid (Figure 8) transporters display novel protein topology and extracellular domain folds that do not fit neatly within current classification schemes that are largely based on transmembrane helix configurations observed in experimental structures of ABC transporters (Thomas and Tampé, 2020; Thomas et al., 2020). Our AlphaFold predictions suggest that there are still novel ABC transporter folds and topologies that have not yet been observed in experimental structures, suggesting that further refinement of ABC transporter classification schemes may be warranted in the near future. Furthermore, the role of new topologies and domains identified in our AlphaFold predictions here remain largely unexplored from both a structural and functional standpoint. Validating that these AlphaFold predictions are accurate through experimental structure determination, and subsequently dissecting the functional roles of novel domains and topologies is likely to provide fruitful insight into the structural and functional diversity of the ABC transporter superfamily.

Another point for consideration is that ABC transporters are inherently dynamic. These transporters change conformations in order to accomplish biological functions. Understanding not only the structure of *B. subtilis* ABC transporters, but also the dynamic conformational landscape of these transporters is paramount to developing a complete molecular understanding for a given transporter. Recent studies have demonstrated that through modulation of multiple sequence alignment depth and other key parameters such as number of recycles or application of structural templates, AlphaFold-2 has the ability to predict multiple conformations of a given membrane transporter (Del Alamo et al., 2022). In our analysis here we utilized the default AlphaFold-2 Multimer pipeline, which results in a single predominant conformation for each of the *B. subtilis* ABC transporter complexes. It is possible that further predictions utilizing different AlphaFold-2 Multimer parameters could yield predictions of these ABC transporter complexes in different conformational states. Moreover, during preparation of this manuscript AlphaFold-3 was released, providing the ability to perform structural predictions of multi-protein complexes in the presence of a limited number of small molecules. Our preliminary studies with AlphaFold-3 in predicting the conformational changes elicited by ATP binding to *B. subtilis* ABC transporters (Figure 8) demonstrate that AlphaFold-3 reliably predicts the ATP binding sites between NBDs of a given ABC transporter complex, and even induced an altered conformation compared to the prediction without added ATP. However, comparison of AlphaFold-3 predicted structures in the presence of ATP revealed significant deviations from experimentally determined structures of the same ABC transporters in an ATP bound conformation. Thus, it appears that further work in reliably predicting not only the structure, but also the conformational landscape of a given ABC transporter complex is warranted. Further developments and code availability will certainly bring about new opportunities to predict the structure of ABC transporters bound to transported substrates and other molecules relevant to function. Such future developments will likely be guided and validated by further experimentally determined structures of ABC transporters in various conformational states and bound to diverse ligands.

With structural predictions now in hand for all ABC transporter complexes in *B. subtilis*, the next challenges moving forward are to validate the predictions that appear to be accurate through experimental means, begin to explore predictions for alternate conformational states, and begin wet-lab structural biology experiments to reveal the structure of complexes that AlphaFold fails to predict reliably. Not only will these directions provide new and deeper insight into the structure and dynamics of ABC transporters in *B. subtilis*, but the results of these experiments will also help to improve future structural predictions of protein complexes that may have otherwise been intractable with currently available software.

## Materials and methods

### Database searches/initial ABC transporter curation

The inventory and assembly of *B. subtilis* ABC transporters previously assembled by Quentin Y. et al. was initially used as a template to inspect the genomic neighborhood, predicted function, and AlphaFold predicted 3-dimensional structure of every plausible ABC transporter. For these initial searches and visual inspection we utilized Subtiwiki (Pedreira et al., 2021), which provided a convenient format to analyze both the genomic context and predicted structure of individual ABC transporter components. From this analysis we compiled a new inventory of ABC transporter complexes in *B. subtilis* complete with updated protein nomenclature, predicted subunit stoichiometries, and biological function. Individual protein amino acid sequences for every component of every ABC transporter complex were compiled into a database that was subsequently used to generate input files for AlphaFold predictions. For the SBP of ABC importers the signal sequence that directs protein translocation to the extracellular space was left intact, and can often be visualized in the resultant structural predictions as an unstructured segment extending off of the NBD. Inclusion of the SBP signal sequence did not impair prediction of any ABC importer structures.

### AlphaFold predictions

AlphaFold predictions were run on a local GPU (NVIDIA RTX-3090) utilizing the LocalColabFold^2^ version of ColabFold (Mirdita et al., 2022). An input.csv file containing a list of all ABC transporter complexes with correct amino acid sequences and stoichiometry was used as an input to predict all structures using the AlphaFold2-Multimer version 2 model (Evans et al., 2022). Predictions were run using the default LocalColabFold settings with 3 prediction recycles and no templates from the PDB or custom PDB templates provided. In order to save computational time, the AlphaFold predictions did not include addition of hydrogens or AMBER relaxation/energy minimization. Output PDB files, pLDDT confidence scores, and predicted aligned error (PAE) were visually inspected on an individual basis for each ABC transporter complex prediction. For each ABC transporter complex AlphaFold generated five atomic models ranked in order of predicted accuracy. For simplicity, only the top ranking model for each ABC transporter complex is displayed in this manuscript.

### Structural similarity search and analysis of unique folds

To identify common domain folds and protein families for regulatory and extracellular domains we employed several bioinformatic tools. The InterPro database (Apweiler et al., 2001) was used to search for protein domains by querying the amino acid sequences corresponding to individual regulatory or extracellular domains. Interpro results were used to identify individual protein domains and their common biological functions. Additionally, the FoldSeek server (van Kempen et al., 2024) was used to identify similar transporters that match the unique topologies found in the extracellular domains of the Type V/VI hybrids.

### Structure visualization and presentation

AlphaFold structure predictions were initially visualized and compared using UCSF Chimera (Pettersen et al., 2004). All figures were generated with UCSF ChimeraX software (Pettersen et al., 2021).

## Data Availability

The datasets presented in this study can be found in online repositories. The names of the repository/repositories and accession number(s) can be found below: https://doi.org/10.5281/zenodo.12806522.

## Footnotes

1
www.alphafoldserver.com

2
https://github.com/YoshitakaMo/localcolabfold
